# Ultrasonography performed by nephrologists: reality and challenges

**DOI:** 10.1590/2175-8239-JBN-2021-E001

**Published:** 2021-02-15

**Authors:** Nordeval Cavalcante Araújo

**Affiliations:** 1 Universidade do Estado do Rio de Janeiro, Departamento de Nefrologia, Rio de Janeiro, RJ, Brasil.

The performance of ultrasound exams by non-radiologists is today a widespread practice around the world. Different medical specialties embraced the method, almost as a propedeutic complement, and incorporated the sonographer into the arsenal of equipment used in the physical examination of patients. In the nephrological environment, the practice was limited to one or two sporadic cases in the 1990s, but today, a large number of professionals use the technique in an ordinary way, as an image method for various procedures, such as renal biopsies and implantation of double-lumen catheters. In at least one particular case, the nephrology unit uses the method for indicating/contraindicating renal biopsies, based on the kidney size and cortical echogenicity relative to the liver, by means of an examination performed by the nephrologist^1^. Centers with such equipment can dramatically reduce the waiting time for the exam. According to Asif, 2003, this time can be only 4.7 days as opposed to 46.5 days, when it depends on the patient’s referral to a radiology department^2^.

From the above, the enthusiasm with which we welcome the article published in this issue of the Brazilian Journal of Nephrology, where Bastos et al. present the results of a survey involving five medical departments from four different states of Brazil^3^. For this survey, aimed at nephrologists, 3,500 e-mails were sent, resulting in 517 responses, making up 15% of the initial sample. Among the most relevant positive data, 78% of public or private institutions have ultrasound equipment and the possibility of use by the nephrologist in 72% of cases can be mentioned. On the other hand, 64% of the participants did not use the method during their training in nephrology, in the vast majority of cases (70%), due to the lack of a staff member with experience in ultrasound use. However, 95% declared an interest in learning the technique. Despite the high percentage of nephrologists interested in it among those who effectively participated, it should be emphasized that this number probably reveals a selection bias, as highlighted by the authors. If we take into account that, in 85% of cases, nephrologists were not even interested in participating, it is very unlikely that the vast majority of those who received the questionnaire would be interested in learning, especially knowing that the questionnaires were sent repeatedly in five different occasions.

Among the difficulties raised by the participants, the lack of time to learn (23%) was only supplanted by the cost of the equipment (26%). However, many nephrologists are uninformed on this subject. According to the American Society of Diagnostic and Interventional Nephrology, the certification of nephrologists to perform and interpret the ultrasound examination of the kidneys and bladder requires a period of just six weeks devoted to training^4^.

In the article, the authors refer to ultrasonography and ultrasound as synonyms, when the former is known to refer to the method of obtaining the image; and the second, the physical principle used. Despite the interchangeable use of the two words in everyday conversations, in a scientific paper such differentiation must be maintained. It would also be better to make it clear, for the reader less familiar with the topic, that the term “point-of-care” ultrasound is the ultrasound examination performed at the bedside. I learned about the case of a colleague who thought that point-of-care ultrasonography incorporated some type of particular physical principle that made the exam distinct, with better image resolution. In fact, the term was borrowed, so to speak, from “point-of-care”, used to refer to the advances in the method of determining glucose in the 1970s, which enabled dosing at the bedside^5^.

The analysis of the compiled data revealed that the most common situations for using the method were due to the classic procedures in nephrology: central vascular access (68%), renal biopsy (58%) and renal evaluation (50%). Perhaps because they were not directly asked about the topic, none of the participants made reference to the use of ultrasound in the evaluation of the transplanted kidney, an important gap, when it is known that ultrasound is the first-line image exam in the immediate postoperative period of patients submitted to kidney transplantation^6^.

In addition to the results and conclusions obtained by the authors, it is worth highlighting the contribution of this survey to the starting undertakings aimed at stimulating the interest of newly graduated doctors in the specialty of nephrology. The inclusion of the technique in the curriculum of medical residency programs, as an integral part of nephrologist’s training, can undoubtedly contribute to reduce the shortage of those interested in the specialty, a phenomenon that has been detected by the Nephrology Societies of other countries^7^^-^ 9. Ultrasonography, with all the inherent virtues of the method, can act as a factor to overcome the “lack of inspirational role model” mentioned by Glassock, 2017^7^. However, this requires a very well coordinated action, which, in my opinion, should be led by the Brazilian Society of Nephrology, in order to reverse the apparent disinterest of the 85% who did not answer the questionnaire. Nephrology sections that already have a well-structured facility in this sense could serve as centers for spreading the idea. Due to the interest in the theme, based on the survey presented, the four centers responsible for the manuscript are on the front line, as well-suited for fulfilling this role.
